# The Association of Fecal Microbiota in Ankylosing Spondylitis Cases with C-Reactive Protein and Erythrocyte Sedimentation Rate

**DOI:** 10.1155/2020/8884324

**Published:** 2020-11-07

**Authors:** Gang Liu, Yonghong Hao, Qiang Yang, Shucai Deng

**Affiliations:** Tianjin Hospital, 406 Jiefangnan Road, Tianjin 300210, China

## Abstract

The purpose of this work was to identify the features of the gut microbiome in cases of ankylosing spondylitis (AS) testing positive for human leukocyte antigen- (HLA-) B27 and healthy controls (HCs) as well as to determine how bacterial populations were correlated with C-reactive protein (CRP) and erythrocyte sedimentation rate (ESR). Fecal DNA extracted from fecal samples from 10 AS cases and 12 HCs was subjected to 16S rRNA gene sequencing. The two research groups did not differ significantly regarding alpha diversity. By comparison to HCs, AS cases displayed a lower relative level of Bacteroidetes (*P* < 0.05), but a higher level of Firmicutes and Verrucomicrobia (*P* < 0.05). Furthermore, the correlation between the specific gut bacteria and ESR or CRP was investigated. At the phylum level, Firmicutes and Verrucomicrobia had a positive association with ESR and CRP, while Bacteroidetes exhibited an inverse correlation with ESR and CRP. Meanwhile, in terms of genus, *Bacteroides* had a positive association with ESR and CRP, whereas *Ruminococcus* and *Parasutterella* had an inverse correlation with ESR and CRP, and *Helicobacter* also displayed an inverse correlation with CRP. Such findings indicated dissimilarities between AS cases and HCs regarding the gut microbiome, as well as the existence of correlations between bacterial populations and both ESR and CRP.

## 1. Introduction

The series of chronic inflammatory joint conditions impacting mainly the spinal, pelvic, and thoracic wall joints of the axial skeleton are known as spondyloarthritis (SpA) [1]. However, it often affects the upper and lower extremities as well, taking the form of arthritis, enthesitis, or dactylitis. Besides its effects on the joints, SpA can manifest as anterior uveitis, psoriasis, inflammatory bowel disease (IBD), such as Crohn's disease (CD) or ulcerative colitis (UC), and urethritis. SpA ranks among the chronic inflammatory rheumatic conditions of greatest frequency as it affects 0.2-1.61% of people [2].

The genes with the greatest predisposition towards ankylosing spondylitis (AS), with more than 90% heritability for AS, are the human leukocyte antigen (HLA) alleles [3]. SpA is often transmitted from one generation to the next, with the risk of acquiring this condition being determined by a number of genetic polymorphisms, especially the HLA-B27 allele belonging to the MHC class I [4]. A close correlation has been established between HLA-B27 and SpA. AS is the radiographic axial subset of SpA, and HLA-B27 is carried by 80-85% of individuals with AS, although the condition evolves in just 5% of those [5]. AS heritability depends on HLA-B27 in a proportion of 20.1%, while an additional 7.38% of the genetic risk is due to a further 113 loci [6].

In cases of genetic predisposition, the condition is also precipitated by the contribution of environmental factors. There is ample proof that the gut microbiome is pathogenically correlated with inflammatory arthritis including SpA [7, 8]. One study on HLA-B27 transgenic rats reported that colitis development was ameliorated when antibiotics targeting anaerobic bacteria, such as metronidazole, or antibiotics with broad action range, such as vancomycin and imipenem, were administered early on [9]. A different study found that caecal inflammation was aggravated when a caecal self-filling blind loop was generated to promote excessive growth of bacteria, while general intestinal inflammation was reduced when the caecum was eliminated from the fecal stream [10]. On the other hand, no significant colon alterations of an inflammatory nature were induced by *E. coli* recolonization, and the progression of colitis was slowed down when probiotic *Lactobacillus rhamnosus* GG was given to HLA-B27 transgenic rats following the administration of antibiotics [11]. A wide range of metabolic activities with direct or indirect connections to the commensal microbiota is susceptible to changes by microbial dysbiosis [12]. It is argued that disease pathogenesis may hinge more on such modifications instead of on differences between species of bacteria.

As suggested above, the microbiota may be involved in SpA pathogenesis. Therefore, a hypothesis can be formulated that the microbiome composition in at-risk individuals might be impacted by HLA-B27. In this work, 16S rRNA analysis was undertaken, with a comparison of fecal samples from adult individuals with AS and healthy controls, to gain more insight into the potential correlation of specific gut dysbiosis and HLA-B27 in AS cases. Another research aim was to determine how the microbiota correlated with C-reactive protein (CRP) and erythrocyte sedimentation rate (ESR), since both CRP and ESR tend to be elevated in AS cases carrying HLA-B27.

## 2. Materials and Methods

The research sample comprised of 22 subjects (Table 1), of whom ten were AS cases and twelve were controls. The modified New York classification criteria for AS informed the employed definition of AS [13]. Informed consent was obtained in writing from all subjects, and the research ethics committee of Tianjin Hospital granted approval for the research procedure. No AS case was on a medication regime of nonsteroidal anti-inflammatory drugs (NSAIDs) or acetaminophen and/or tramadol.

### 2.1. Analysis of CRP, ESR, and HLA-B27

The Boster enzyme-linked immunosorbent assay kit was employed to measure the level of CRP in the blood plasma of the research subjects, while the Westergren technique was applied in keeping with the International Council for Standardization in Hematology to determine ESR (ml/hr) [14]. The HLA-B27 was tested with flow cytofluorometry according to the methods described by Albrecht and Muller [15].

### 2.2. 16S rRNA Gene Sequencing and Analysis

Feces were sampled from each of the 22 research subjects to enable DNA analysis. The Qiagen QIAamp DNA Stool Mini Kit was employed for the extraction of total genomic DNA from the fecal samples, while the primers 338 F 5′-ACTCCTACGGGAGGCAGCA-3′ and 806 R 5′-GGACTACHVGGGTWTCTAAT-3′ were used for amplification of the V3-V4 hypervariable region of the bacterial 16S rRNA gene. The Qiagen Gel Extraction Kit (Qiagen, Germany) facilitated the mixing in ratios of identical density and the purification of PCR products. The TruSeq® DNA PCR-Free Sample Preparation Kit (Illumina, USA) was used to produce sequence libraries, with the addition of index codes. The Qubit@ 2.0 Fluorometer (Thermo Scientific) and the Agilent Bioanalyzer 2100 system permitted evaluation of the quality of the libraries. The Illumina HiSeq2500 platform was employed for library sequencing, with production of 250-bp paired-end reads, which were allocated to samples according to their singular barcode, followed by truncation through excision of the barcode and primer sequences. The integration of paired-end reads was achieved via FLASH51, which was geared towards fusing paired-end reads in response to the overlapping of at least a few of the reads with the read produced from the other end of an identical DNA fragment. The name given to the trimmed sequences was raw tags. The QIIME quality control process was applied for filtering those tags to yield uncontaminated tags of high quality [16]. The UCHIME algorithm allowed a comparison between the tags and the reference database for the purpose of identifying chimeric sequences that were subsequently eliminated [17, 18]. This procedure eventually generated the effective tags.

Uparse software (Uparse v7.0.1001) was employed to analyze the sequences. The sequences that were similar in proportion greater than 97% were allocated to identical OTUs [19], and a sequence representative for every OUT was chosen for additional annotation. The RDP classifier algorithm was applied to extract taxonomic information from the GreenGene Database regarding the representative sequences [20]. Normalization of OUT abundance was done according to the sequence count in the sample with the lowest number of sequences. The normalized data were then employed to analyze alpha and beta diversity. Observed-species, Chao1, Shannon, Simpson, ACE, and Good-coverage were the alpha diversity indices that were used for the analysis of the complexity of species diversity. QIIME (version 1.7.0) and R software (version 2.15.3) were, respectively, employed for calculation and visualization of each index.

### 2.3. Statistical Analysis

SPSS (version 22.0, IBM SPSS Inc., USA) was employed for statistical analysis, with the expression of the generated values taking the form of mean ± SD. The intergroup statistical analysis was performed via Student's *t*-test, while Spearman's rank correlation analysis was performed to determine how gut microbiota and CRP or ESR were correlated. Differences of statistical significance were reflected by a *P* value of less than 0.05.

## 3. Results

### 3.1. Elevated CPR and ESR in AS Cases

**Figure 1:**
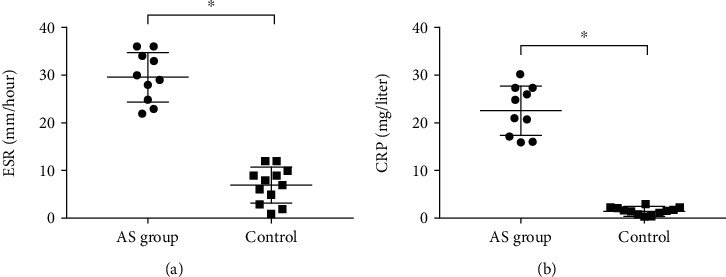
The ESR and CRP levels in the AS and control groups. ^∗^*P* < 0.05.

Figure 1 provides details about the research sample. Compared to the HCs, the AS cases had significantly higher levels of both CRP (Figure 1(a)) and ESR (Figure 1(b)) (*P* < 0.05).

### 3.2. Phylum-Level Microbiota Differences

**Figure 2:**
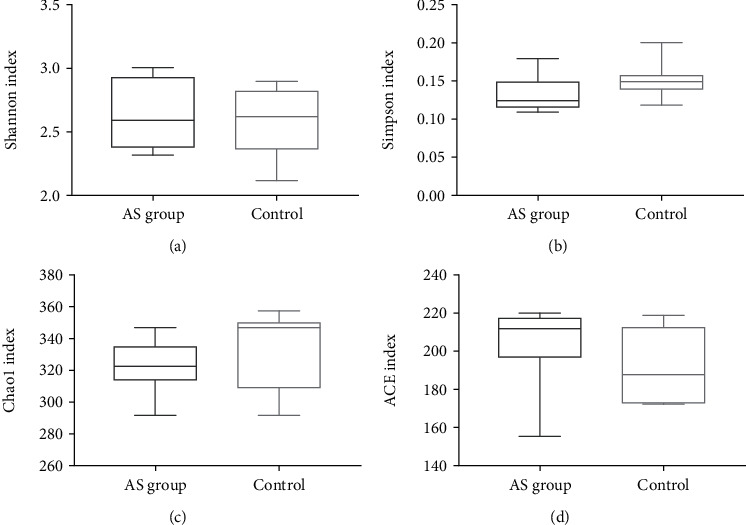
The diversity indices associated with populations of bacteria present in feces. Box plots reflect microbiome diversity discrepancies between the AS cases and HCs as revealed by the Shannon index (a), Simpson index (b), Chao1 index (c), and ACE index (d).

Diversity indices were applied to assess how diverse the fecal microbes were. The AS cases and HCs did not differ in terms of the Shannon, Simpson, Chao1, and ACE indices (Figure 2).

**Figure 3:**
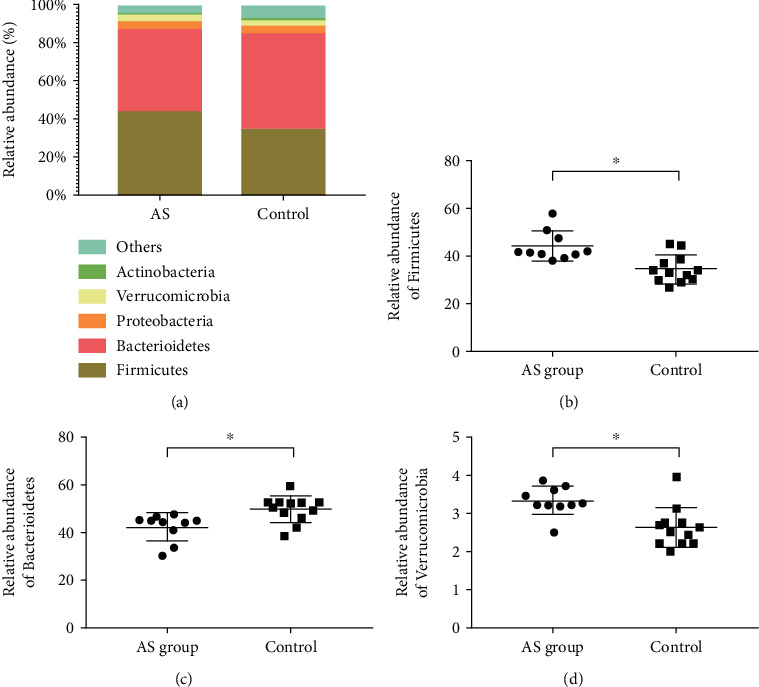
Phylum-level examination of microbiome composition. (a) The relative abundance of fecal microbes at the phylum level. Intergroup comparison of relative abundance of (b) Bacteroidetes, (c) Firmicutes, and (d) Verrucomicrobia. ^∗^*P* < 0.05.

At the level of phylum, Firmicutes (44.08% in AS cases and 34.62% in HCs), Bacteroidetes (42.62% in AS cases and 50.10% in HCs), Proteobacteria (4.10% in AS cases and 4.01% in HCs), Verrucomicrobia (3.35% in AS cases and 2.65% in HCs), and Actinobacteria (1.01% in AS cases and 0.94% in HCs) were the main bacterial populations in feces (Figure 3). Furthermore, by comparison to HCs, AS cases showed a reduction in Bacteroidetes relative abundance, but an increase in the relative abundance of Firmicutes and Verrucomicrobia (*P* < 0.05).

### 3.3. Genus-Level Microbiota Discrepancies

**Figure 4:**
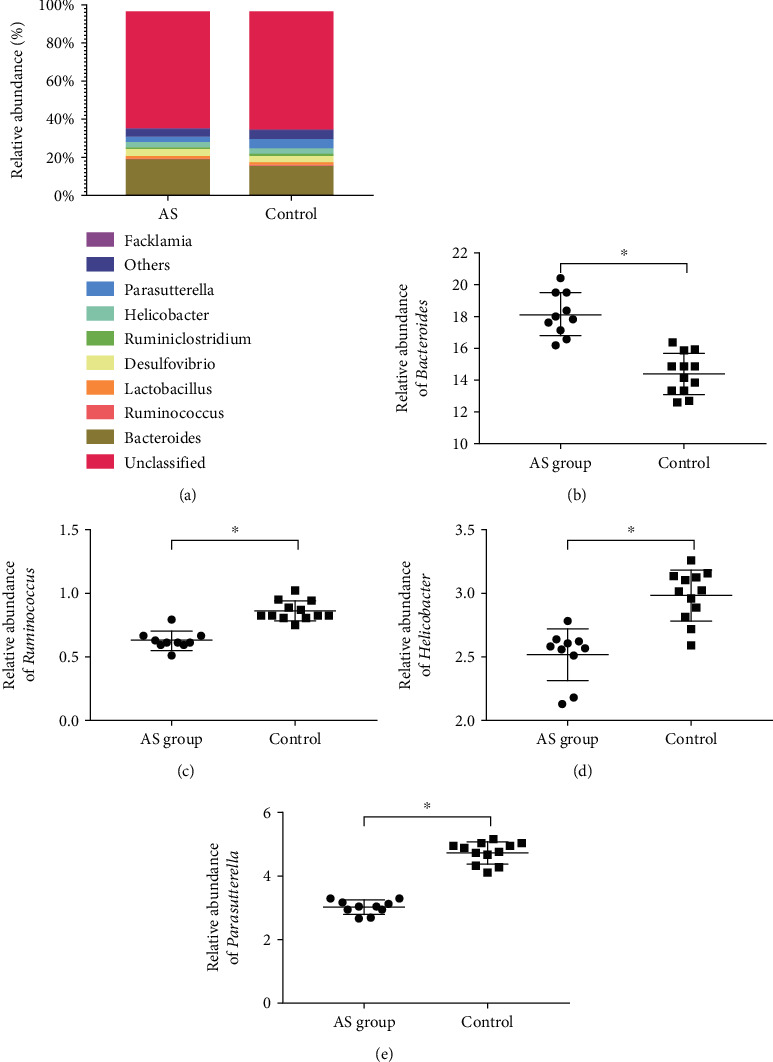
Genus-level examination of microbiota composition. (a) The relative abundance of fecal microbes at the genus level. Intergroup comparison of relative abundance of (b) Bacteroides, (c) Ruminococcus, (d) Helicobacter, and (e) Parasutterella. ^∗^*P* < 0.05.

Figure 4 illustrates the genus-level abundance of eight major microbes. At the genus level, *Bacteroides* (18.14% in AS cases and 14.40% in HCs), *Ruminococcus* (0.63% in AS cases and 0.86% in HCs), *Lactobacillus* (1.19% in AS cases and 1.30% in HCs), *Desulfovibrio* (3.16% in AS cases and 2.93% in HCs), *Ruminiclostridium* (1.22% in AS cases and 1.19% in HCs), *Helicobacter* (2.52% in AS cases and 2.99% in HCs), *Parasutterella* (3.03% in AS cases and 4.75% in HCs), and *Facklamia* (0.66% in AS cases and 0.59% in HCs) were the main bacterial genus in feces (Figure 3). Furthermore, by comparison to HCs, AS cases showed a reduction in relative abundance of *Ruminococcus*, *Helicobacter*, and *Parasutterella* genus, but an increase in the relative abundance of *Bacteroides* (*P* < 0.05).

### 3.4. Microbiota Associations with CPR and ESR

**Figure 5:**
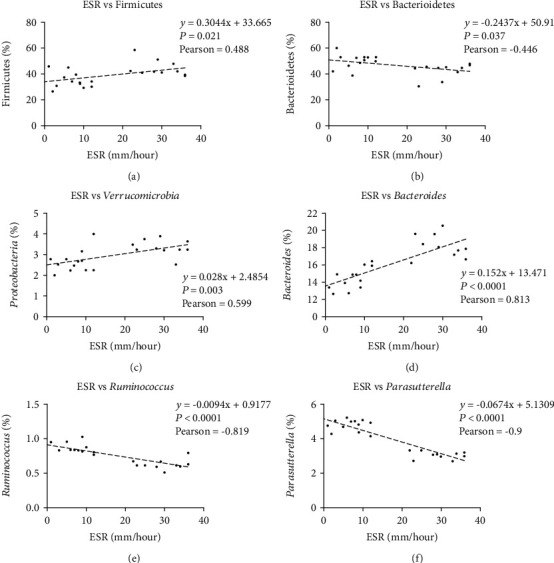
The association of microbiota with ESR. (a) Positive correlation between Bacteroidetes abundance and ESR; (b) Inverse correlation between Firmicutes and ESR; (c) Positive correlation between Verrucomicrobia and ESR; (d) Positive correlation between *Bacteroides* and ESR; (e) Inverse correlation between *Ruminococcus* and ESR; (f) Positive correlation between *Parasutterella* and ESR.

To determine how the microbiota and ESR were correlated at the level of phylum and genus, Spearman's correlation analysis was carried out, revealing a positive correlation of Firmicutes (Figure 5(a)) and Verrucomicrobia (Figure 5(c)) with ESR at phylum level and direct correlation between *Bacteroides* (Figure 5(d)) and ESR, but inverse correlation of *Ruminococcus* (Figure 5(e)) and *Parasutterella* (Figure 5(f)) with ESR at genus level.

**Figure 6:**
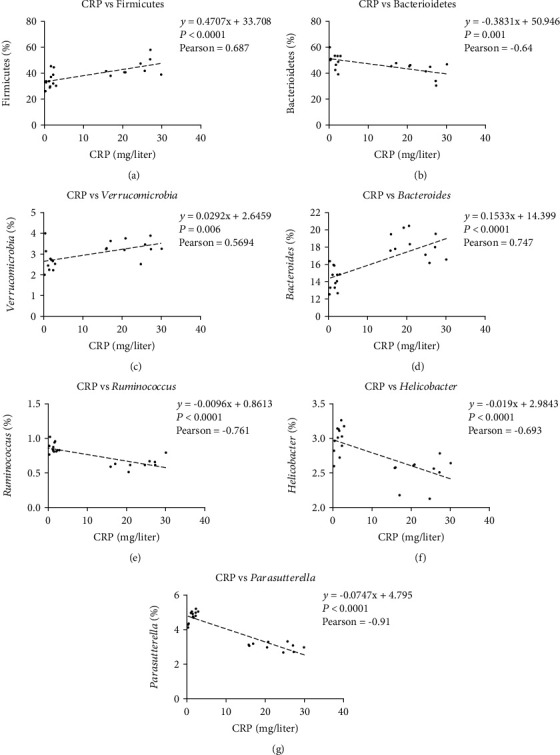
The association of microbiota with CRP. (a) Positive correlation between Bacteroidetes abundance and CRP; (b) Inverse correlation between Firmicutes and CRP; (c) Positive correlation between Verrucomicrobia and CRP; (d) Positive correlation between *Bacteroides* and CRP; (e) Inverse correlation between *Ruminococcus* and CRP; (f) Inverse correlation between *Helicobacter* and CRP; (g) Positive correlation between *Parasutterella* and CRP.

To determine how the microbiota and CRP were correlated at the level of phylum and genus, Spearman's correlation analysis was carried out, revealing a positive correlation of Firmicutes (Figure 6(a)) and Verrucomicrobia (Figure 6(c)) with CRP at phylum level and positive correlation between *Bacteroides* (Figure 6(d)) and CRP, but inverse correlation of *Ruminococcus* (Figure 6(e)), *Helicobacter* (Figure 6(f)), and *Parasutterella* (Figure 6(g)) with CRP at genus level.

## 4. Discussion

Classified as SpA “prototype”, AS represents a chronic, progressive inflammatory autoimmune condition that affects primarily the sacroiliac joints and the vertebral column [21]. The present work conducted 16S rRNA sequence analysis of fecal samples to detect the profile intestinal dysbiosis in the microbiome of individuals with AS.

The contribution of the gut microbiota to AS is widely acknowledged, but knowledge remains limited as to the exact form taken by that contribution [8]. The general consensus is that both genetic and environmental factors underpin AS development [22]. In the context of investigations of disease etiology, the human gut microbiome should be afforded due consideration, given the likely correlation between indigenous gut bacterial populations and AS. Nevertheless, a comprehensive explanation of AS etiology is yet to be formulated, and the identification of potential causative factors is ongoing. Interest in human microbiome mapping has risen considerably in the recent ten years [23], and it has become possible to determine the exact species making up the microbiota owing to the introduction of molecular-based techniques (e.g., metagenomics), which are more advanced than standard culture-based techniques. In spite of this, there are still several important aspects that need to be addressed, including the involvement of the microbiota in AS and the mechanism of microbiome impact on immune response as well as local and systemic inflammation.

The present work provides convincing proof that there are differences in gut microbiome among cases with and without AS. AS cases were found to have a markedly greater abundance of *Bacteroides*, but a lower abundance of *Ruminococcus*, *Helicobacter*, and *Parasutterella*. Such changes might have a regulating effect on innate and adaptive immunity, thus contributing to AS development [24]. This work compared cases with and without AS in terms of microbiome density and abundance to improve knowledge of how the gut microbiome varied between different populations as well as how the microbiota-host cross-talk occurred. *Klebsiella pneumoniae* and *Bacteroides vulgatus* are among the bacteria that have been identified to be essential for AS pathogenesis [25]. Nevertheless, the amount of bacteria is typically associated with causal immune response rather than infection. According to research conducted on animal models, *Bacteroides* contribute to the occurrence of inflammation in peripheral joint or intestinal conditions. In line with earlier studies [8, 26], this work confirmed that *Bacteroides* abundance was greater in AS cases than in HCs. Furthermore, studies have demonstrated that both IBD and arthritis developed as a result of a rise in the level of HLA-B27 in transgenic rat gut that reflected the presence of *Bacteroides vulgatus* [26, 27].

The existence of a correlation between some constituents of the microbiota and ESR or CRP was confirmed by correlation analysis. It was thus deduced that AS pathogenesis depends significantly on the gut microbiota. Hence, future studies should profile the species constituting the AS-related microbiota and shed light on how the gut microbiome contributes to AS progression.

Additional aspects worth researching include the extent to which genetic factors underpin alterations in gut microbiota and what implications this has for the general microbiome functionality in AS cases and its impact on immune response and inflammation. Further attention should also be given to the hypothesis that AS is triggered by HLA-B27 through its impact on the gut microbiome, which was formulated in light of the identified correlation between HLA-B27 and AS. By expanding knowledge about such aspects, comprehension of AS development can be improved.

## Figures and Tables

**Table 1:** The traits of the AS cases and HCs at the point of biopsy^∗^.

Subject ID	Age, years	Sex	Disease duration, years	ESR, mm/hour	CRP, mg/liter	HLA–B27
AS-01	44	M	7	23	27.25	Positive
AS-02	45	F	5	33	24.65	Positive
AS-03	59	F	4	30	20.52	Positive
AS-04	46	F	2	22	25.75	Positive
AS-05	48	M	6	25	20.80	Positive
AS-06	52	M	7	36	17.02	Positive
AS-07	48	F	5	36	29.96	Positive
AS-08	57	M	6	29	27.14	Positive
AS-09	40	M	3	34	15.85	Positive
AS-10	53	M	7	28	15.98	Positive
HC-01	35	F	—	12	0.45	Negative
HC-02	47	M	—	6	2.43	Negative
HC-03	39	F	—	9	2.26	Negative
HC-04	54	M	—	10	1.38	Negative
HC-05	52	M	—	1	1.67	Negative
HC-06	43	F	—	2	0.23	Negative
HC-07	51	M	—	9	0.52	Negative
HC-08	47	F	—	5	1.80	Negative
HC-09	47	F	—	3	2.95	Negative
HC-10	44	F	—	12	1.56	Negative
HC-11	46	M	—	7	1.22	Negative
HC-12	43	M	—	8	2.33	Negative

## Data Availability

The original data used to support the findings of this study are available from the corresponding author upon request.
